# Neighbourhood Walkability and Its Influence on Physical Activity and Cardiometabolic Disease: A Cross-Sectional Study in a Caribbean Small Island Developing State

**DOI:** 10.7759/cureus.44060

**Published:** 2023-08-24

**Authors:** Kern D Rocke, Christina Howitt, Jenna Panter, Mark Tully, Ian Hambleton

**Affiliations:** 1 George Alleyne Chronic Disease Research Centre, Caribbean Institute for Health Research, The University of the West Indies, Bridgetown, BRB; 2 Medical Research Council (MRC) Epidemiology Unit, Centre for Diet & Activity Research (CEDAR), University of Cambridge, Cambridge, GBR; 3 Institute of Mental Health Sciences, School of Health Sciences, Ulster University, Newtownabbey, GBR

**Keywords:** health of the nation survey, recent physical activity questionnaire, 10-year cardiovascular risk, urban planning, small island developing states, caribbean, cardiovascular disease, physical activity, built environment

## Abstract

Introduction

Cities and neighborhoods may provide opportunities for population-level environmental interventions to reduce physical inactivity and cardiometabolic risk. In this study, we describe the association between neighborhood walkability, physical activity (PA), and cardiometabolic outcomes, by linking data from a nationally representative survey of adults (25 years and older) collected in 2012-2013 with spatial data on built environment features in Barbados.

Methods

We estimated a walkability index for 45 neighborhoods using objectively measured built environment features (residential density, street connectivity, and land use mix). We used the Recent Physical Activity Questionnaire to capture time spent in outdoor walking, active commuting, moderate-to-vigorous PA (MVPA), and total PA. Our primary cardiometabolic outcome was a predicted 10-year cardiovascular risk (CVD) score, estimated using the American College of Cardiology/American Heart Association pooled cohort equation. Our secondary cardiometabolic outcomes were hypertension and diabetes. We explored the effect of neighborhood walkability on PA and cardiometabolic outcomes using several multivariable regression models (tobit and linear and logistic multi-level mixed effects), with the model choice depending on the structure of the outcome.

Results

The average time spent walking weekly for any purpose among participants was 75 minutes/week, time spent on active commuting was 15 minutes/week, and MVPA was 221 minutes/week. We estimated that the average 10-year CVD risk in the study population was 11.7% (95%CI 10.9-12.5). Our confounder-adjusted analyses showed positive linear relationships between neighborhood walkability and each PA outcome (p<0.05 in all cases), and a negative relationship between walkability and predicted 10-year CVD risk (p<0.001).

Conclusion

In our setting, adults residing in higher walkability neighborhoods spent more time engaged in PA, had a lower predicted 10-year CVD risk, and were less likely to have diabetes. Urban planners may consider shorter-term interventions, such as those on a microscale, which may provide additional ways to increase activity in a mostly fixed macroscale environment.

## Introduction

Since the turn of the century, cardiovascular disease (CVD) has been the leading cause of morbidity and mortality globally [1,2]. Across the Caribbean, high cardiovascular burdens similar to developed countries have been reported [3,4]. To address this burden, countries have focused on the primary prevention of cardiometabolic diseases, including CVD and diabetes, with the aim of reducing the prevalence of associated risk factors. Insufficient physical activity (PA) has been established as a key modifiable risk factor for cardiometabolic disease [5]. A recent review of longitudinal evidence reported that adults achieving the WHO recommended amount of PA had a 17% lower risk of a CVD event, 26% lower risk of developing type 2 diabetes, and 23% lower likelihood of CVD mortality [6]. Despite these potential benefits, engagement in regular physical activity continues to decrease globally [7]. Self-reported data from Caribbean national surveys suggest that between one-quarter and one-half of adults are physically inactive [8], with the majority of countries higher than the global average of 27.5% [9]. Moreover, when physical activity is measured objectively as reported by Howitt et al., the rates of inactivity increase further [10].

Physical activity interventions targeting individuals have produced inconsistent results in the medium and long term, and efforts to address the burden of physical inactivity have turned towards redesigning the built environment to offer more opportunities for physical activity [11,12]. Cities and neighborhoods may provide opportunities for population-level environmental interventions to reduce physical inactivity and cardiometabolic risk [13]. Neighborhoods that support pedestrian activities, such as those with well-connected streets and destinations within comfortable walking distances, have been linked with higher levels of PA [14-17] and lower levels of adverse health outcomes, including CVD and diabetes [18-21]. Recently, the concept of walkability has received increased attention in the global literature [22,23]. Walkability can be thought of as a neighborhood’s capacity to support an active lifestyle or encourage PA of any type (active commuting, leisure time, or moderate-to-vigorous PA (MVPA)). Frank et al. combined three fundamental aspects of walkability (land-use mix, residential density, and street connectivity) to create a walkability index [24], which has been widely utilized to study the relationship between walkability, PA, and health outcomes [19,20,25].

While the importance of the relationship between walkability, physical activity, and cardiometabolic diseases has been previously described and documented, most of this work has been performed in North America and Europe. There are no published studies examining these relationships in the Caribbean and very few in other Small Island Developing States (SIDS) [26]. The effect of walkability on health may be influenced by the different patterns of urbanization in SIDS. Their small geographic size and limited land resources commonly result in unusual urban structures, often a single urban center with connections to outlying communities, for example along a coastal perimeter. Therefore, we aimed to describe the association between neighborhood walkability, physical activity, and cardiometabolic outcomes among adults from a Caribbean SIDS setting.

## Materials and methods

Study design and population

Our physical activity and cardiovascular information was based on a nationally representative cross-sectional survey of adults aged 25 years and over living in Barbados, the Health of the Nation (HoTN). Data were collected for 1234 consenting participants between 2011 and 2013 on health conditions and behaviors (including questionnaire-assessed PA), as well as sociodemographic information. Details of the sampling, recruitment, and data collection methods have been previously described [27]. Written informed consent was obtained from all survey participants. Complete data on physical activity were available from all participants for all PA outcomes (overall walking, active commuting, MVPA, and total PA). In addition, complete data on 10-year CVD risk estimates was available for 1110 (90%) participants. The study received ethical approval from the Research Ethics Committee of the University of the West Indies, Cave Hill Campus, Barbados, and Barbados Ministry of Health and Wellness.

Neighborhood definition

Our study did not have available individual address-level data from the HoTN for geocoding thus we chose to define our neighborhoods using administrative boundaries. Defining neighborhoods remains a methodological challenge [28,29]. In Barbados, there is no clear distinction of what defines a neighborhood. For this study, we considered enumeration districts (EDs) as neighborhoods. EDs, similar to census tracts in the United States, are the smallest geographic unit into which Barbados is divided for census and survey data collection. The average size of EDs in Barbados is 0.74 km^2^ with an average population size of 388 residents. The HoTN study used EDs as its primary sampling unit and we used it as a proxy for defining neighborhoods in our study. Each participant was assigned the ED of their primary residence. The HoTN study had a total of 45 EDs with an average of 27 study participants in each ED.

Neighborhood walkability 

**Figure 1:**
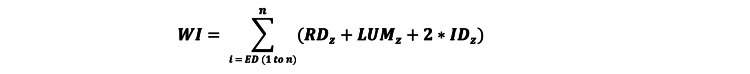
Walkability Index Equation WI = Walkability Index; RD= Residential Density; LUM= Land Use Mix; ID= 3-way Intersection density; z= Z-score; ED= Enumeration District

We estimated a neighborhood walkability index at the ED level using an adjustment to methods (exclusion of retail floor) highlighted by Frank et al. [24], and this adjustment has been used in previous studies [20,30-32]. Our index uses the following built environment features captured after 2010: residential density, street connectivity (intersection density), and land use mix (which characterizes urban design, density, and diversity) (Appendix 1; [24,32-35]). Details on how each component was estimated can be found in the appendices. We normalized each component into z-scores before calculating the neighborhood walkability as the sum of the z-scores of the three built environment measures. Figure 1 gives the walkability index equation. 

The resulting walkability index was re-scaled for ease of interpretation, with the re-scaled index ranging from 0-100 where 0 represented the ED with the lowest and 100 represented the ED with the highest walkability. We estimated the neighborhood walkability index island-wide (n=583 EDs), which included all HoTN study neighborhoods (n=45 EDs). The quintiles of these estimates were visually mapped to describe the spatial distribution of the index in our study setting.

Physical activity and walking

Physical activity was determined using the Recent Physical Activity Questionnaire (RPAQ), which assesses PA over the past four weeks. Details on the use of this tool in this population have been previously described [10]. For the current study, we determined the weekly time spent on the following PA outcomes: outdoor walking (walking for transport, pleasure, and exercise), active commuting (walking and biking for transport), MVPA (combination of activities that get individuals moving fast or strenuous enough to burn three or more times the energy per minute compared to when they are at rest [36]) (Appendix 2), and total PA (any reported activity, regardless of context or intensity). 

Cardiometabolic outcomes

The cardiometabolic outcomes for our study were hypertension, diabetes, and 10-year CVD risk. We defined hypertension using the WHO STEPS criteria of either systolic blood pressure of ≥ 140 mmHg or diastolic blood pressure of ≥ 90 mmHg or if the participant was currently on medication to treat hypertension. Diabetes was defined as either a glycated hemoglobin (HbA1C) ≥48 mmol/mol or if the participant was currently on medication to treat diabetes or self-reported that they had been diagnosed with diabetes.

We estimated 10-year CVD risk using the American College of Cardiology (ACC)/American Heart Association (AHA) Pooled Cohort Equation risk score in adults in the age group of 40-79 years [37]. We included the following variables in our calculations: age, gender, ethnicity, self-reported smoking status, total cholesterol, systolic and diastolic blood pressure, hypertension treatment status, and diabetes. Total cholesterol was obtained from laboratory testing. Ten-year CVD risk estimates are presented as percentages. Higher estimate scores equate to a greater chance of an atherosclerotic CVD event (heart attack or stroke) during the following 10 years. We created three binary variables with categories (greater than 7.5%, 10%, and 20%) based on clinical cut-offs for treatment interventions [38]. This categorization method has been used in a previous study in Caribbean populations [39].

Statistical analysis

All univariate analyses were survey-weighted to account for the survey sampling design and participant non-response, and to match the age and gender distribution of the Barbados population based on the census closest in time to the survey results (the 2010 national census) [40]. We created walkability categories using tertiles of the walkability index, representing low, moderate, and high walkability, then present stratified descriptive statistics by walkability categories for continuous variables as means and categorical variables as percentages with their associated 95% confidence intervals (CIs). 

To account for the multi-level nature of the data (1,234 participants within 45 EDs, 45 EDs within 10 parishes), we used multi-level regression models to examine the relationship between our study outcomes and neighborhood walkability. These administrative units were as used for the national population census [40]. Our multi-level regression models incorporated random effects at the parish and ED level. 

To examine the relationship between weekly time spent engaging in PA and neighborhood walkability, we selected a multi-level tobit regression model. This model allowed us to left-censor the 46% of participants who reported zero minutes of activity. This approach has been used in other transport and walkability studies [41-43]. For our model, we report minutes of weekly activity as our main association measure. To examine the relationship between 10-year CVD risk (a continuous measure) and neighborhood walkability, we selected a multi-level linear regression model. For this model, we report a 10-year risk percentage estimate as our main association measure. Lastly, to examine the relationship between neighborhood walkability and our binary outcomes (10-year CVD risk categories, hypertension, and diabetes) we selected a multi-level logistic regression model. We report odds ratios as our main measure of associations for these models. The observed outcome estimate change in each model was associated with a 10-point increase in neighborhood walkability.

We present univariate models showing unadjusted estimates between our main outcome and neighborhood walkability. Our multivariable models controlled for individual and neighborhood-level confounders. Potential confounders were added to our regression models using a forward iterative approach whereby variables were retained based on the Akaike information criterion (AIC) (with lower AIC values suggesting improved model fit). Our final list of included confounders were age, gender, ethnicity, highest level of education, body mass index (BMI), car ownership, and neighborhood socioeconomic status (SES). Our neighborhood SES score was developed using household data from the 2010 national census at the ED level. Details on the variables included and the method of estimating the SES score can be found in Appendices 3-5.

We present exact p-values for all models and assumed that p-values less than 0.05 were statistically significant. As well as presenting p-values, we preferentially presented 95% CIs for our association estimates to recognize the role of uncertainty in our modeling. Data were analyzed using Stata Statistical Software: Release 17 (2021; StataCorp LLC, College Station, Texas, United Stated) [44].

## Results

Descriptive analysis

**Figure 2:**
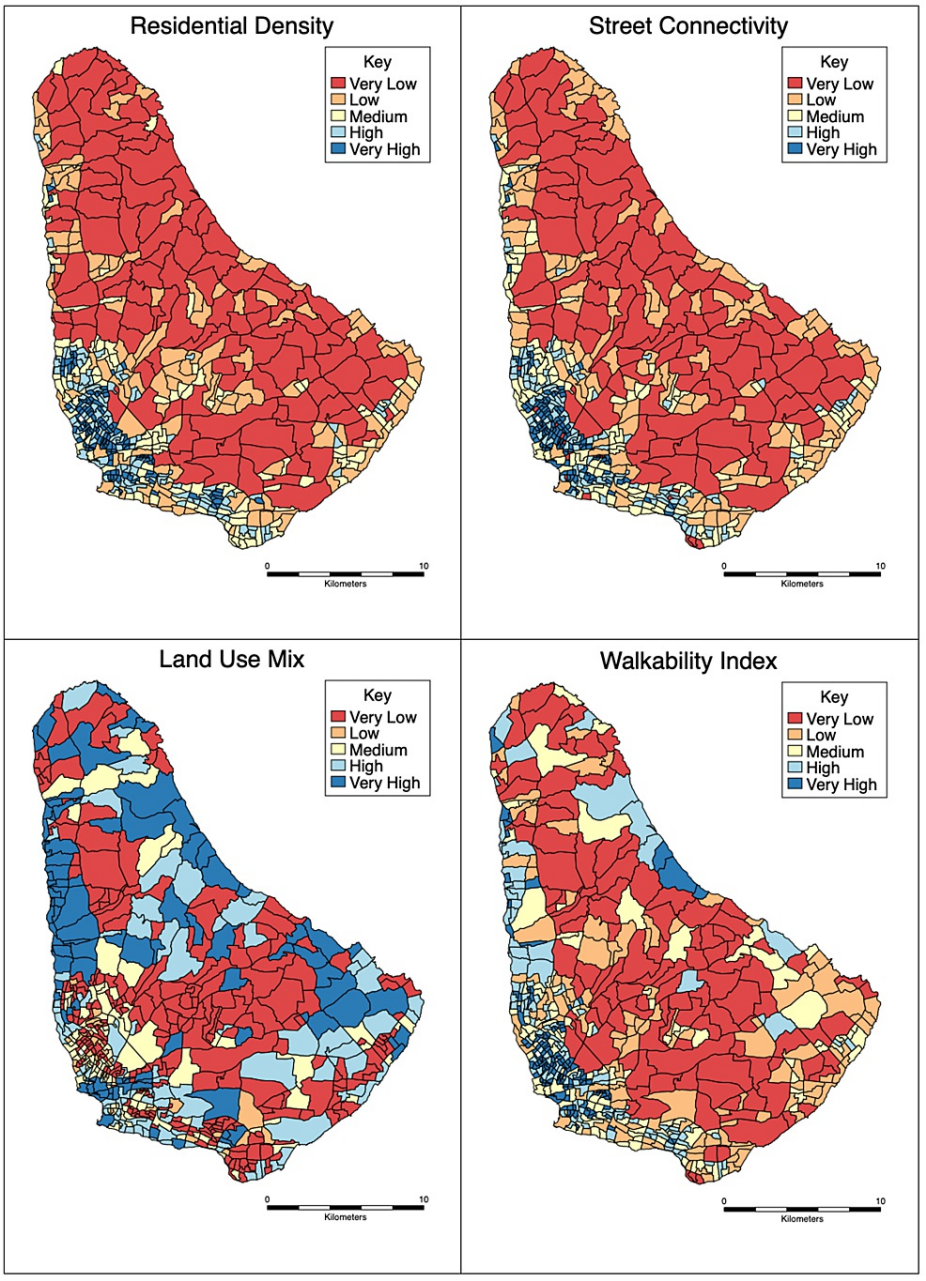
Spatial Distribution of Neighbourhood Built Environment Features and Walkability in Barbados

**Figure 3:**
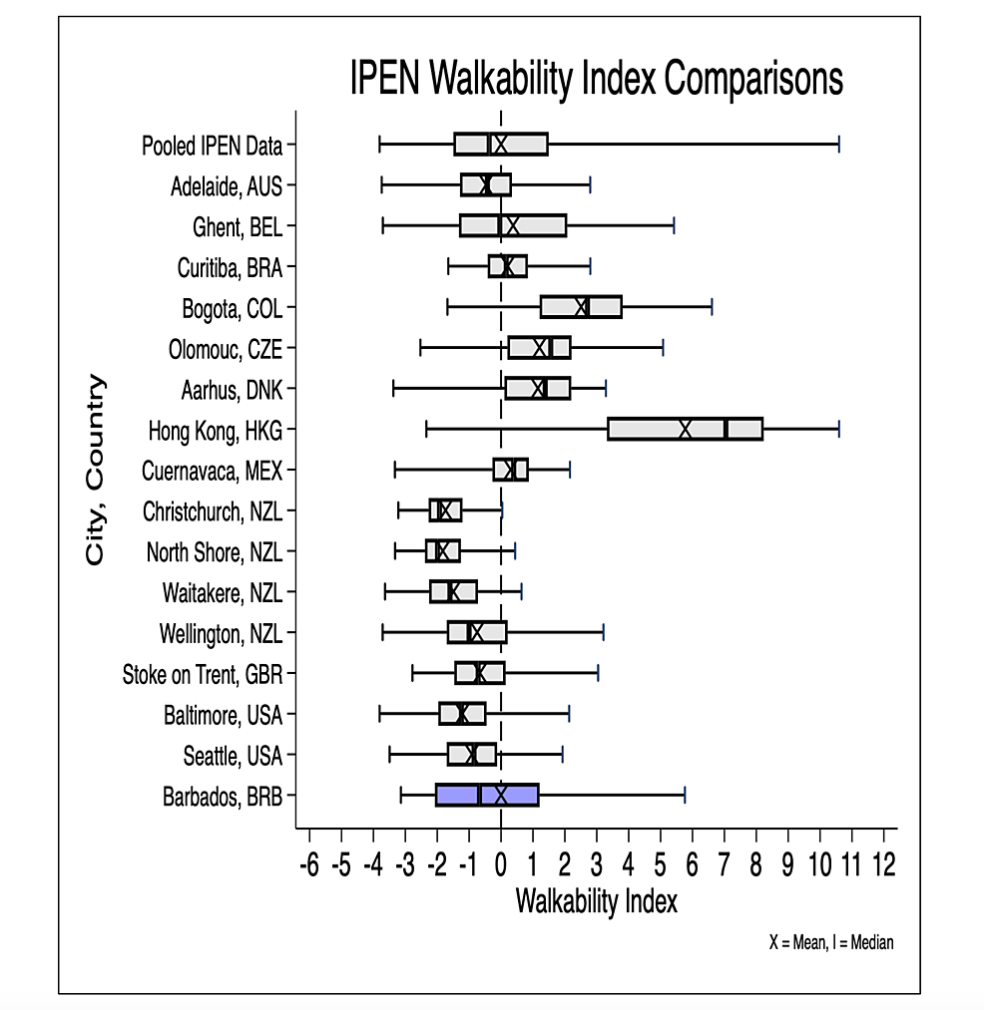
Comparison of IPEN Walkability Indices Across IPEN Study Sites With Sites in Barbados IPEN: International Physical Activity and Environment Network; NZL: New Zealand; AUS: Australia; BEL: Belgium; BRA: Brazil; COL: Colombia; CZE: Czechia; DNK: Denmark; HKG: Hong Kong; MEX: Mexico; GBR: Great Britain; USA: United States of America; BRB: Barbados

The spatial distribution of the neighborhood walkability index and its components in Barbados is presented in Figure 2. Broadly, coastal areas had higher walkability and inland areas had lower walkability. We compared our estimated neighborhood walkability index to indices previously estimated among settings within the International Physical Activity and Built Environment (IPEN) study, which used an identical walkability methodology [34]. We found our estimates for neighborhood walkability were within the range of the IPEN study pooled multi-country estimates (Figure 3). We estimated the national neighborhood walkability index for Barbados to be 16.5 (range: 0-100) while for the study neighborhoods, it was estimated to be 17 (range: 1.1-52.4). 

The study population characteristics by walkability levels are presented in Table 1. The weekly average time spent walking for any purpose among participants was 75 minutes/week, time spent on active commuting was 15 minutes/week, MVPA was 221 minutes/week, and total activity was 3721 minutes/week. We estimated that the average 10-year CVD risk in the study population was 11.7% (95%CI: 10.9, 12.5). 

**Table 1:** Study Population Characteristics by Neighborhood Walkability Categories Estimates are presented as mean/percentage (95% CI); Self-reported from the RPAQ CVD: cardiovascular risk; MVPA: moderate-to-vigorous physical activity; SES: socioeconomic status; BMI: body mass index; ED: enumeration district; RPAQ: Recent Physical Activity Questionnaire

Characteristic	Neighborhood Walkability		
	Low n=331 (15 EDs)	Moderate n=332 (16 EDs)	High n=302 (14 EDs)
Age (years)	46.1 (43.5, 48.7)	46.4 (44.5, 48.3)	47.3 (45.6, 49.0)
Gender			
Female	47.2 (41.5, 53)	53.2 (44.8, 61.7)	48.5 (40.7, 56.2)
Male	52.8 (47, 58.5)	46.8 (38.3, 55.2)	51.5 (43.8, 59.3)
Education			
Primary/Less	9.9 (4.9, 15.0)	7.0 (3.4, 10.5)	16.0 (9.9, 22.1)
Secondary	54.5 (46.7, 62.4)	44.7 (38.6, 50.7)	42.2 (34.7, 49.6)
Tertiary	35.5 (27.2, 43.8)	48.4 (41.8, 54.9)	41.9 (33.3, 50.4)
BMI (kg/m^2^)	29.1 (28.1, 30.1)	28.0 (27.3, 28.6)	27.9 (26.9, 28.8)
Car ownership	56.7 (50.6, 62.8)	61.6 (54.9, 68.4)	47.5 (39.8, 55.3)
Overall walking (mins/week)	64.3 (43.5, 85.2)	64.7 (48.5, 81.0)	99.0 (68.2, 129.8)
Active commuting (minutes/week)	7.9 (3.6, 12.2)	13.2 (6.6, 19.8)	26.7 (12.6, 40.8)
MVPA (minutes/week)	220.6 (187.9, 253.3)	198.6 (164.7, 232.4)	245.5 (187.6, 303.4)
Total Activity (minute/week)	3684.4 (3472.8, 3895.9)	3820 (3616.1, 4024)	3654.3 (3435.9, 3872.7)
Hypertension	31.4 (26.7, 36.1)	28.8 (22.6, 34.9)	30.5 (23.4, 37.5)
Diabetes	12.7 (8.4, 17)	11.9 (6.3, 17.5)	10.7 (7.9, 13.5)
10-year CVD Risk Score	12.0 (10.7, 13.4)	11.2 (9.6, 12.6)	11.9 (10.6, 13.3)

Associations between neighborhood walkability and physical activity

The results of the unadjusted and adjusted analyses for each PA outcome are presented in Table 2. We found in our multivariable analyses adjusted for known confounders that participants living in higher walkability neighborhoods spent more time engaging in outdoor walking and active commuting compared to participants from lower walkability neighborhoods. A 10% increase in neighborhood walkability was significantly associated with an increase in weekly time spent walking, active commuting, and in MVPA. 

**Table 2:** Association Between Neighborhood Walkability and Time Spent on Weekly Physical Activity Tobit regression used by left censoring 0 minutes of activity; Multi-level model used (Parish and enumeration districts); Multivariable model adjusted for sex, age, ethnicity, education, car ownership, smoking, BMI, hypertension status, diabetes status, neighborhood SES MVPA: moderate-to-vigorous physical activity; SES: socioeconomic status

Outcome (mins/week)	Unadjusted Model		Multivariable Adjusted Model	
	β (95% CI)	p-value	β (95% CI)	p-value
Overall walking (minutes/week)	18.5 (6.0, 31.0)	0.004	17.6 (2.3, 32.8)	0.024
Active Commuting (minutes/week)	54.5 (21.0, 88.0)	0.001	50.2 (19.1, 81.3)	0.002
MVPA (minutes/week)	10.3 (3.6, 17)	0.003	17.6 (7.6, 27.5)	0.001
Total activity (minutes/week)	-5.0 (-56.9, 47)	0.852	76.9 (28.5, 125.2)	0.002

A 10% increase in neighborhood walkability was associated with a weekly increase of 17.6 minutes of overall walking (95%CI: 2.3, 32.8; p=0.024); 50.2 minutes of active commuting (95% CI: 19.1, 81.3; p=0.002); 17.6 minutes of MVPA (95%CI: 5.8, 29.4; p=0.004). Additionally, multivariable models showed a significant association with total activity (β = 76.9; 95%CI: 28.5, 125.2; p=0.002).

Associations between neighborhood walkability and CVD outcomes

The results of the unadjusted and multivariable-adjusted analyses for each CVD outcome are presented in Table 3. In the adjusted multivariable models, we observed a statistically significant negative association between neighborhood walkability and 10-year CVD risk in participants aged 40-79 years. A 10% increment in neighborhood walkability resulted in a 0.57% reduction in CVD risk (95%CI: -0.88, -0.27; p=<0.001). This may suggest that cardiovascular risk may be a distal risk factor compared to PA. We also observed that participants had a statistically significant lower probability of having a 10-year CVD risk score of 20% and above for every 10% increase in neighborhood walkability (OR= 0.87; 95%CI: 0.77, 0.99; p=0.031). This may suggest that the effect of neighborhood walkability with increasing 10-year CVD risk may become stronger with higher individual risk score estimates. In addition, we observed a statistically significant lower likelihood of diabetes with increased neighborhood walkability but not with hypertension. 

**Table 3:** Association Between Neighborhood Walkability and 10-Year CVD Risk with its Associated Risk Factors ^a^ Multi-level mixed effects linear regression model; ^b^ Multi-level logistic regression model Models adjusted for age, sex, education, ethnicity, car ownership, neighborhood SES, BMI (when not used as an outcome), time spent on total activity, smoking CVD: cardiovascular disease; β: percentage CVD risk score

	Unadjusted Model		Multivariable Adjusted Model	
Outcome	β^a^ (95% CI)	p-value	β^a^ (95% CI)	p-value
10-year CVD Risk (%)	0.08 (-0.45, 0.61)	0.780	-0.57 (-0.88, -0.27)	<0.001
	OR^b^ (95% CI)	p-value	OR^b^ (95% CI)	p-value
10-year CVD Risk (≥7.5%)	1.06 (0.92, 1.22)	0.387	0.96 (0.77, 1.20)	0.720
10-year CVD Risk (≥10%)	1.03 (0.95, 1.11)	0.535	0.83 (0.68, 1.02)	0.070
10-year CVD Risk (≥20%)	1.01 (0.94, 1.09)	0.815	0.87 (0.77, 0.99)	0.031
Hypertension	0.99 (0.91, 1.08)	0.856	0.94 (0.85, 1.04)	0.250
Diabetes	0.90 (0.82, 0.98)	0.017	0.81 (0.75, 0.88)	<0.001

## Discussion

In our sample of adults in a Caribbean SIDS, we found that higher neighborhood walkability was associated with increased outdoor walking, active commuting, and total PA. Furthermore, we observed that people who live in neighborhoods of higher walkability have a lower predicted 10-year risk of having a CVD event; this reduced risk, although small, could be relevant from a public health perspective. Despite the modest decreases in 10-year CVD risk, these small shifts at the individual level can accumulate to more significant reductions in CVD and non-communicable disease outcomes at the neighborhood and national levels. To our knowledge, our findings are the first in the Caribbean to link neighborhood walkability with PA and CVD risk.

We found that a small increase (10%) in neighborhood walkability was associated with an additional 18 minutes of MVPA per week, which equates to 12% of the recommended WHO guidelines. A 10% increase in walkability was also associated with at least 50 more minutes of active commuting and total activity per week. These increases are sufficient to impact health. The most recent guidelines published by the WHO emphasize that increasing activity levels by even small amounts is beneficial, and that even if the minimum recommendations are not met, some activity is better than none [45]. Our findings are in line with studies in industrialized settings, which demonstrate that living in a more walkable neighborhood is associated with increased levels of outdoor walking, active commuting, MVPA, and overall PA [31,46-49]. A recent cross-sectional study in the Netherlands by Lam and colleagues found a positive association between neighborhood walkability and walking trips for transport-related and essential shopping purposes [43,50]. A study conducted in Brazil by Siqueira-Reis and colleagues found that neighborhood walkability was positively associated with walking for transport and leisure-time MVPA [51]. Overall, our findings from a Caribbean SID add to the global evidence base that supports the potential role of walkability in promoting PA.

Our findings suggest that living in more walkable neighborhoods could also have benefits for cardiovascular health. We found that neighborhood walkability was associated with a statistically significant reduction in CVD risk. Although the evidence examining this relationship is limited globally, our findings are consistent with published evidence. Howell and colleagues found that Canadian residents living in less walkable neighborhoods had a higher 10-year CVD risk [19]. Coffee and colleagues reported that the probability of developing a CVD risk factor decreased by 6% with each 10-point increase in walkability [52]. The concept of walkability as an upstream determinant of cardiovascular health is likely to be the result of a walkable environment leading to increased PA. This in turn may limit age-related weight gain, reduce body fat, waist circumference, and BMI, and may improve blood pressure and insulin resistance, all factors that drive cardiovascular risk during later life [21,53,54]. Our findings suggest that enhancing neighborhood walkability should be considered as an enabling factor in designing environmental interventions aimed at reducing the burden of CVD.

Caribbean settings have similarities that set them apart from larger industrialized nations, including their small size and regularly blurred distinction between urban and rural community developments, the predominance of neighborhoods near or along coastal areas, and a tropical climate with high heat and rainfall [55]. These factors potentially play an important role in the idiosyncrasies of how well Caribbean neighborhoods support walking. Nonetheless, the concepts that define macroscale walkability, i.e. the interconnectedness of routes and the proximity to popular destinations, are sufficiently broad that we hypothesized that they would still influence walking behavior, despite differences in other environmental factors. We therefore adapted the methods frequently used to define objectively measured walkability [24], and have shown that walkability does influence activity levels in our setting. Making neighborhoods more activity-friendly could therefore be part of the solution to the non-communicable disease pandemic in the Caribbean. Due to resource constraints and limited planned development, changing walkability at the macroscale level would be challenging. A promising avenue for future work may therefore be to assess the impact of the microscale built environment on physical activity. The microscale built environment refers to pedestrian-level features, such as sidewalks and crossings, which are more amenable to low-cost intervention than macroscale characteristics.

Study strengths included utilizing objectively measured BE features using internationally recognized methods to estimate neighborhood walkability, allowing for comparisons to other settings. Secondly, we used lab-based measures to estimate 10-year CVD risk. Lastly, we examined a nationally representative population of Barbados, which may allow for further extrapolation of our findings to the wider public. Our study has important limitations, which should be considered when interpreting our results. Our analyses were cross-sectional; thus, we are unable to establish a temporal relationship between neighborhood walkability and our outcomes (physical activity and 10-year CVD risk). We did not measure neighborhood self-selection, which may be an important confounder highlighting differences in behaviors based on their self-selection status [56,57]. The estimated 10-year CVD risk scoring was based on an algorithm that was developed for North American populations and has not been validated in a Caribbean setting; however, we chose the AHA/ACC CVD risk scoring algorithm due to its widespread use clinically in the Caribbean [58]. Finally, our study did not objectively measure physical activity behavior; rather, we relied on self-reported estimates from the RPAQ tool, and thus our physical activity estimates could have been overestimated as previously reported [10]. 

## Conclusions

Individuals living in more walkable neighborhoods had higher levels of physical activity and a lower predicted risk of developing a cardiovascular event over the next 10 years. Furthermore, despite several environmental idiosyncrasies of SIDS, our study shows similarities with other parts of the world, lending weight to the concept that walkability is a design principle that applies across different settings. Given the rise of urbanization and increasing trends in highly concentrated populous communities, walkability and its potential impact on health within neighborhoods should be considered within urban planning policies and public health interventions, with improving the built environment considered a priority. However, any improvements to the built environment (increased mixed land use, connectivity, and residential density) require substantial time and investment from all key stakeholders.

## Appendices

Appendix 1: Walkability index components

Residential Density

a) Description: Ratio of the number of residents or dwellings within each ED to the land area of the ED

b) Computation: Number of residential dwellings/ED land area (km^2^)

Street Connectivity/Intersection Density

a) Ratio between the number of true road and footpath intersections (three or more intersections) to the land area of the ED. Intersections for highways are excluded. Higher densities corresponded to more direct paths between destinations. The inclusion of footpaths was considered as this was seen as routes individuals use to access destinations in the Caribbean.

b) Computation: Sum of road and footpath intersections(>=3 way intersections) / ED land area (km2)

Land Use Mix

a) Description: Diversity of land uses within each ED. Five land use types were considered as has been used in previous studies [24,33,34]. These land uses are residential, retail, entertainment, office, and institutional (e.g., schools and community institutions). Land uses not fitting these categories are omitted from the analysis. There is no consideration for spatial locations of urban objects thus this estimation takes into account statistical methods rather than geospatial techniques. Most studies and the IPEN Adult and Adolescent studies developing walkability indices have used the entropy index as their main estimator for land use mix [24,32,35].

b) Computation:  $LUM= - \sum_{j=1}^{k} P^jln(P^j)/ln(k)$

Appendix 2: MVPA

Moderate PA

Walking for exercise, walking for pleasure, cycling for pleasure, mowing the lawn, watering the lawn/garden, digging/shoveling/chopping wood, weeding/pruning, other types of aerobics, exercise with weights, conditioning exercises, push-ups/sit-ups/pull-ups, dancing, tennis/badminton, table tennis, golf, cricket, netball/volleyball/basketball, fishing, horse-riding, sailing, windsurfing, surfing

Vigorous PA

Swimming, hiking, racing (cycling and running), jogging, high-impact aerobics, jogging, squash, road tennis, football, rugby, hockey, rowing/kayaking, martial arts, boxing, wrestling

Appendix 3: Description of confounding variables

Age: Participant age in years

Sex: Participant sex (Male; Female)

Ethnicity: Participant ethnicity (Black; Non-Black)

Education: Highest level of education (Primary; Secondary; Tertiary)

BMI: Participant body mass index (kg/m^2^)

Smoke: Smoking status (No; Current smoker)

Car ownership: Usage of a private car for transport

Hypertension status: Systolic blood pressure>140 and Diastolic blood pressure>90 or the participant is currently on prescribed medication for hypertension

Diabetes status: Fasting glucose >7mmol/L or HbA1C > 48 mmol/mol, or participant is currently on prescribed medication for diabetes

Neighborhood SES index: Estimated at the ED level using data from the 2010 national census and principal component analysis

Appendix 4: Neighbourhood SES index estimation

Census socioeconomic data from each enumeration district of Barbados was obtained from the Barbados Statistical Service (BSS). Data obtained from the BSS represented data collected from the 2010 national census of Barbados. All data was checked and cleaned prior to analyses. Socioeconomic data that was captured in the census included age, income, education, occupation, household size, main activities, type of work activity, crime, ethnicity, house tenure, immigration status, presence of household amenities, and vehicle ownership. Data were stratified by gender and both male and female for all areas.

However, these analyses only utilized estimates for both males and females. Proportion estimates for all variables were computed by ED for further analyses. Normality checks of all variables were conducted using the Shapiro-Wilks test for normality. Variables found to violate the assumption of normal distribution were transformed into the logarithmic version. Bivariable Pearson correlations were conducted for all variables. Once significant correlations were observed (p<0.05), these were then inputted into the principal component analysis model. This technique is a data reduction technique commonly used to derive socioeconomic scores by aggregating factors of socioeconomic status (income, education, occupation, household dwellings, etc.) into components that represent broadly the overall SES of a study population. Rotation was conducted using orthogonal transformation (varimax and oblique rotation) to allow for greater interpretability of components. Sensitivity analysis was conducted to explore variation in area-level scores using different variable retention methods (variables included; rotation method and Least Absolute Shrinkage and Selection Operator (LASSO) regression). Our results showed that using the PCA with varimax rotation approach with 18 variables was most appropriate. 

Appendix 5: List of variables used to estimate neighborhood SES index

Non-black population; Median age; Young age dependency; Old age dependency; Mean household size; Housing tenure -  Owner; Housing tenure - Renting; Single Mother; Less than secondary education; Tertiary education; Median income; Low income ($0-49999); High income (>$150000); Unemployment; Liveborn children >5; Crime victim; Management occupations; Professional occupations; Technical occupations; Non-technical occupations; Household structure: number of rooms; Household structure: number of bedrooms; Household structure: number of bathrooms; Toilet presence; Vehicle Ownership; No Vehicle Ownership; Stove; Refrigerator; Microwave; Computer; Radio; Television; Washing Machine; Household lighting: Electricity
